# Self-monitoring urinary salt excretion device can be used for controlling hypertension for developing countries

**DOI:** 10.1186/s40885-019-0109-9

**Published:** 2019-03-15

**Authors:** Yasmin Jahan, Michiko Moriyama, Md Moshiur Rahman, Atiqur Rahman

**Affiliations:** 1 Graduate School of Biomedical & Health Sciences, Hiroshima University, Hiroshima, Japan; 2 Department of Social and Welfare Studies, Linkoping University, Linkoping, Sweden

**Keywords:** Salt monitoring device, Urinary salt excretion, Dietary salt intake, Developing countries

## Abstract

Restriction of dietary salt is widely recommended in the management of hypertension, but assessment of individual salt intake has drawn little attention. Monitoring nutritional salt intake through sodium excretion has been popular, because the main route for sodium (Na) excretion is through the urine. Nonetheless, direct measurement of dietary salt intake is time consuming and lacks accuracy. To collect a 24-h urine and measure the content is difficult method for most patients. In this review paper, we would like to explore the usefulness of measuring urinary salt excretion by using a self-monitoring device at home. Measuring daily overnight urine by the self-monitoring device at home will be useful for the management of hypertension suitable for each individual. From the recent increase of processed foods, the term “salt intake” would not accurately be equal to “sodium intake”. Devices measuring urinary sodium excretion have been developed and evaluated on their accuracy and correlation with sodium intake. They must be handy, simple and capable of measuring large populations to be useful for monitoring of daily salt intake and to guide salt restriction as well as the long-term effects by dietary salt intake.

## Background

Hypertension (HTN) is one of the foremost non-communicable diseases (NCDs) in the world [1], which significantly contributes to the burden of cardiovascular diseases (CVDs), stroke, kidney failure, disability, and premature death [2–4]. According to the World Health Organization (WHO), about 17 million deaths occur worldwide due to CVDs, of which hypertension alone accounts for 9.4 million deaths [5, 6] and 80% of the CVD-related deaths occurred in the developing countries [7]. Not only that, hypertension has become a significant health concern in the Asian region, affecting more than 35% of the adult population [8].

For developing and onset of HTN, excess dietary salt intake plays a significant role [9, 10]. As we know that restriction of salt intake is important lifestyle modification and widely recommended for the management of hypertension. According to World Health Organization’s recommendations restrict salt intake to 5 g/day or less [11]. Nevertheless, many people are still consuming more salt than recommended. Most people are not aware how much salt they consume from day to day. Moreover, encouragement or education to reduce salt intake seems insufficient in comparison to that present in developed countries [12]. However, changing behavior to a reduction in salt intake is not easily done in practice. It is necessary to strengthen the hearsay knowledge and relayed massage among the high-risk persons so that a strong perception will develop, leading to behavioral change [13]. One of the most important ways to educate people about salt restriction is to accurately evaluate their daily salt intake. While the guidelines of the World Health Organization and International Society of Hypertension (WHO/ISH) stated that counseling by a skilled nutritionist and monitoring of the urinary Na level are necessary in most cases, but they did not mention specific methods for these purposes [14].

Nonetheless, some medical institutions estimate salt intake by using a 24 h urine sample. But repeated measurements to evaluate daily salt intake are quite difficult. Further, salt intake estimation using spot urine [15] or the second morning urine, although easy, also require the measurement of sodium and creatinine concentrations, and thus, cannot be performed daily [16]. So, it is important to develop some strategies for educating people about the need to reduce their salt intake. One strategy could be the self-monitoring of urinary salt excretion; Thus, a means by which people could monitor daily salt intake by themselves might be effective for controlling Blood Pressure (BP). A device that may be useful with this strategy is a self-monitoring device that was recently developed and estimates 24 h urinary salt excretion from overnight urine samples [17]. This was recommended as a useful strategy by the Working Group for Dietary Salt Reduction of the Japanese Society of Hypertension [18]. This study aimed to explore the usefulness of this urinary salt self-monitoring device for hypertensive individuals regarding their levels of salt intake and the dangers of excessive salt use.

## Reason to choose this device

Excess intake of dietary salt is estimated to be one of the leading risks to health worldwide. Major national and international health organizations, along with many governments around the world, have called for reductions in the consumption of dietary salt. In developed and developing countries, salt added during food processing is the dominant source of salt and largely outside of the direct control of individuals [12, 19]. However, salt intake is assessed using nutrition surveys. But in this paper, we want to discuss about such device which a person can measure his/her urinary salt excretion at their home. Some medical institutions estimate salt intake by using a 24 h urine sample [20, 21] but repeated measurements to evaluate daily salt intake are quite difficult. Contrariwise, a self-monitoring device can estimate 24 h urinary salt excretion from overnight urine samples [22]; Salinometer, a self-monitoring device can estimate 24 h urinary salt excretion from overnight urine samples; moreover, individuals can use this device at home and thus obtain daily measurements. Yamasue K et al. (2006) reported the self- monitoring device estimates 24 h salt excretion from overnight urine samples by using the following formula: Y = 1.95X + 4.5 g/day, where Y is the estimated 24 h urinary salt excretion and X is the sodium content of the overnight urine sample [17]. Another study Yasutake k et al. (2016) stated overnight sodium content (X) can be converted to 24 h salt excretion (Y) by Y = 5.76(X)^0.53^ g/day. Careful comparison of overnight with 24 h urine collection methods have developed the above converting formula making these devices more reliable [23]. There is another Self-monitoring Device for measuring Urinary Sodium-to-Potassium Ratio (HEU-001F, OMRON Healthcare Co., Ltd., Kyoto, Japan) [24]. By this device we can see the ratio of Na and K. But by KME 03 device we only can measure urinary salt excretion. In this paper we are trying to reduce the prevalence of hypertension with its related complications by reducing salt consumption, so that we are focusing on only urinary salt excretion salinometer device.

## Methods of using self-monitoring device

A KME-03 salinity checker (developed by KOUNO ME Institute, Kanagawa, Japan) might be used to determine the estimated daily salt intake. This device consists of a 1 L urine cup and an electrical device with volume and conductivity sensors. The volume sensor, which consists of 50 small resistant chips, measures overnight urine volume in the cup. The conductivity sensor consists of two gold-plated nickel metal plates. A temperature compensating circuit is used because conductivity changes with temperature. In this method, urine have to collect for 24 h, and salt intake will be assessed by determining the urinary Na excretion. This method is considered to be reliable and is used in many clinical and epidemiological studies [9]. These are the following steps for measurement of urinary salt excretion:

1. Void completely and discard the urine before going to bed.
2. Collect the overnight urine (including the first urine after awakening) in the 1 L urine cup. About 8 h urine is preferable.
3. Set the salt monitor and push the source button Daily salt excretion is shown on the panel. (after about 15 s).
4. Remove the salt monitor from the cup and void the urine. Wipe the urine on the sensor by tissue or toilet paper.
5. Put the clean water in the cup and discard the water. Add water again.
6. Set the salt monitor. After cleaning the censor by the water, wipe the water on the sensor by tissue or toilet paper. (Never wash the device except sensor).
7. Discard the water of the cup. Set the salt monitor until next morning.
8. Next day, remove the salt monitor from the cup and collect the overnight urine again [25].

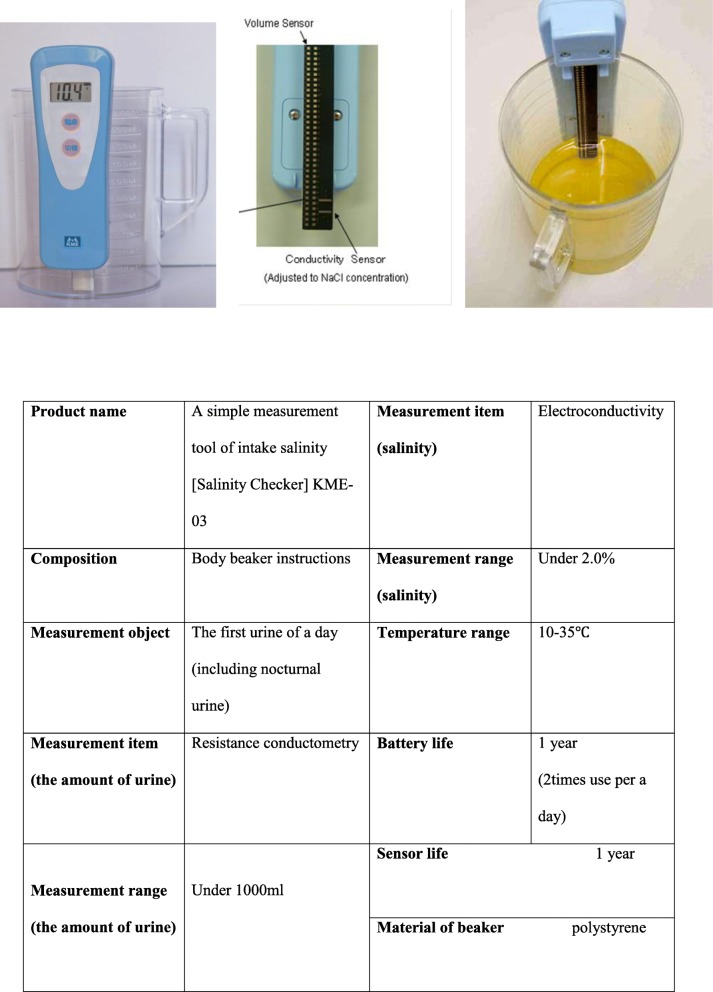

## Discussion

Numerous studies have shown that some people are not sensitive to salt. Sensitivity and resistance to the effects of salt have been evaluated by the degree of blood pressure change between high-salt intake and low-salt diet [26–28]. Recent reports suggest that morning surge is closely related to incidence of cardiovascular and cerebrovascular diseases [29, 30]. A 24 h period is necessary to capture the marked diurnal variation in sodium, chloride and water excretion. Electrolyte excretion in healthy individuals reaches a maximum at or before midday, and a minimum at night towards the end of sleep [31]. In addition, blood pressure and salt intake differ in individuals and on different days.

Evaluation of sodium intake by urine sodium level needs precautions such as ruling out some diseases (hypertension, diabetes, cancer) and other related factors. Measurement of 24 h urine sodium excretion has been used as a golden standard for evaluating sodium intake. However, 24 h urine samples are inconvenient, costly for a large population. Alternative methods, such as spot, overnight, daily, timed urine samples have been taken into concern whether it can reflect sodium intake of individuals as well as large populations. There were studies showing correlation between spot and 24 h urine collection [32]. Nevertheless, sodium excretion is not constant throughout the day, and intra-individual variation has to be concerned. A study suggested that at least a week of overnight samples is necessary to evaluate urinary sodium excretion level [33]. Repeated measurements can increase the reliability of spot urine analyses [34]. But spot-urine evaluation is not always useless, because there are studies where evidence can be discovered by this simple, easier method, especially in clinical practice [35].

However, several studies had been conducted for urinary salt monitor from 24 h urinary sample or from overnight urine sample. Among them Yasutake K et al. (2016) found that the estimated 24 h urinary salt excretion from the overnight urine samples reflected salt intake from the previous day. The average 24 h urinary salt excretion (the ratio of urinary salt excretion to salt intake of the previous day) estimated from the overnight urine samples was as follows: 8.01 ± 1.15 g (0.73 ± 0.11) on days 2–6, 5.86 ± 0.85 g (1.01 ± 0.15) on days 7–9, 9.69 ± 1.64 g (0.74 ± 0.13) on days 10–12, 6.51 ± 1.56 g (1.03 ± 0.25) on day 13, 8.60 ± 3.25 g (0.71 ± 0.14) on day 14, and 6.28 ± 1.31 (1.05 ± 0.22) on day 15 [23]. Concerning the validity of the self-monitoring device, Yamasue et al. (2006) reported a significant correlation between i) salt excretion values estimated using overnight urine and a self-monitoring device, and ii) salt excretion measured by 24-h urine collection (r = 0.72, *P* < 0.001) [17]. Ohta et al. (2009) also reported that salt excretion measured with a self-monitoring device correlated well with that determined by 24 h home urine evaluation in hypertensive subjects (r = 0.63, *P* < 0.01. Mean estimated daily salt intake of all participants was 9.9 g/day [36], which was higher than the recommended salt intake (5 g/day) [37]. In fact, mean salt intake is estimated more than 11.7 g/day in many Asian countries, and salt intake reduction remains a public health challenge in this particular region [10].

However, there is strong and consistent evidence from animal studies, clinical trials and epidemiological data both within and across populations implicating high salt intake as an important risk factor for high BP among both hypertensive and normotensive individuals [38–45]; high salt intake is also associated with increased risk of future CHD and stroke [10, 46–48]. There are differences between developed and developing countries with regard to dietary sources of salt. In some developing countries, where sodium in the diet comes from salt added during cooking. These countries are amid an epidemiological transition with increasing rates of chronic diseases including hypertension, CVD and its related complications [49, 50]. There is therefore the opportunity for timely community based, context-specific initiatives to limit the amount of salt added to food by individuals [51]. It is also essential, given the increasing westernization of these cultures, to encourage low levels of salt content in processed foods, which are likely to become increasingly available in the developing world. Such initiatives are needed to tackle the forecasted epidemic of CVD in developing countries.

Detection and treatment of hypertension is a major component of cardiovascular risk reduction. However, the underlying causes of hypertension, such as poor diet, are often left ‘untreated’. Patients with drug treated hypertension are at higher risk of CVD than individuals with normal BP. Similarly, amongst drug-treated hypertensives, those with unhealthy lifestyle are at a higher risk of CVD than those with healthy lifestyle [52, 53]. There is also evidence that reduced salt intake in treated individuals may reduce the number and dose of drugs or obviate the need for treatment. Thus, it would be useful to measure salt intake with the new salt monitor and to examine the relationship between blood pressure and salt intake for a lengthy period. One of the major limitations of this device was it was not compared to other standard method. So further research can be done regarding this.

## Conclusions

Historical improvement of devices on salt-intake are still on their way to prevent devastating cardiovascular, cerebrovascular diseases. Recent development of devices that measure urinary salt excretion correlate well with sodium intake making them promising tools for preventing these diseases. First, they will evoke awareness of high-salt intake in regions where high-salt intake prevails. Second, these tools will be helpful in evaluating salt intake on various upcoming trials on high salt intake-induced diseases. The devices must be developed to evoke awareness of deadly consequences of high-salt intake in regions where high-salt intake prevails. Cardiovascular diseases are not only deadly, but once diagnosed; also have undesirable impact on the individual as well as health economics.

Although there are several methods for the assessment of salt intake, the precise determination of salt intake in individual patients is difficult. Reliable methods are difficult to perform, and simpler methods are less reliable. However, the assessment of salt intake is strongly recommended, because it is useful for informing patients of their salt intake and conducting salt restriction. In addition, further interventional study can be approached for established this home monitoring device. Moreover, public health initiatives, in tandem with efforts by the food industry, are urgently needed to lower salt consumption and consequently lower CVD burden and increase life expectancy. Such public health approaches can be simple, low-cost and effective.
